# Correlation Between Rotator Cuff Tear in Thai Urban Elderly Population and Vitamin D Deficiency

**DOI:** 10.7759/cureus.54986

**Published:** 2024-02-26

**Authors:** Sutee Thaveepunsan, Ekkalak Kosasaeng, Yupadee Fusakul, Sitthiphong Suwannaphisit

**Affiliations:** 1 Department of Orthopaedics, Faculty of Medicine Vajira Hospital, Navamindradhiraj University, Bangkok, THA; 2 Department of Rehabilitation, Vajira Hospital, Navamindradhiraj University, Bangkok, THA; 3 Department of Orthopaedics/Hand and Microsurgery, Faculty of Medicine Vajira Hospital, Navamindradhiraj University, Bangkok, THA

**Keywords:** fat infiltration, rotator cuff muscle, articular cartilage, vitamin d level, rotator cuff tear

## Abstract

Background

The benefits of vitamin D encompass the augmentation of rotator cuff healing, the enhancement of bone mineral density (BMD), and the fortification of skeletal muscle strength. The vitamin D deficiency in Asian countries appears to be more severe compared to their Western counterparts. This study aims to ascertain the relationship between rotator cuff tears and vitamin D levels in the urban Thai elderly demographic. Our hypothesis posits that vitamin D deficiency will exhibit an association with the occurrence of rotator cuff tears.

Materials and methods

A prospective clinical trial conducted at a single tertiary was carried out to assess the patients experiencing shoulder pain who were aged 60 years or older. All participants were tested of blood specimens for calciferol concentration and magnetic resonance imaging (MRI). The duration between blood sample collection and magnetic resonance imaging (MRI) did not exceed a two-week window. The assessment of fatty degeneration in the supraspinatus, infraspinatus, and subscapularis muscles, as well as tear dimensions and cartilage thickness, was conducted using magnetic resonance imaging within the outpatient clinic.

Results

The analysis of serum vitamin D levels within a cohort comprising 59 subjects produced significant observations, indicating that 20.03% of the participants manifested a deficiency in vitamin D and 44.07% exhibited insufficiency in vitamin D levels. There was no observed correlation between serum vitamin D levels and various patient parameters, including age (P = .99), body mass index (P = 0.31), tear size (P = 0.41), cartilage thickness at different locations on the humeral head (superior, middle, inferior, and total) (P = 0.31, 0.40, 0.26, 0.20, respectively), degree of fatty infiltration of the rotator cuff (P = 0.81), and severity of the rotator cuff condition (P = 0.13). A significant positive correlation was established between rotator cuff tear size and both the severity of the rotator cuff condition (P < 0.001) and the degree of fatty infiltration of the cuff (P < 0.001).

Conclusion

A negative correlation is observed between serum vitamin D levels and various parameters, including tear size, fatty infiltration, cartilage thickness, and the severity of rotator cuff tears within the elderly urban Thai population. To affirm these findings, it is imperative to conduct additional research and integrate vitamin D assessments into the management strategies for aging populations with rotator cuff conditions.

## Introduction

Vitamin D plays a crucial role in regulating bone health, aiding in fracture healing, and contributing to soft tissue functionality [1]. Changes in vitamin D levels have a significant impact on the musculoskeletal system, potentially leading to adverse effects like articular cartilage degeneration when vitamin D levels are insufficient [2-4]. Particularly noteworthy is the effectiveness of vitamin D supplementation in the elderly population. Studies have shown its ability to improve both upper and lower body muscular strength, reducing the risk of fragile fractures and falls [5].

Recent scholarly investigations have delved into the complex role of vitamin D in the pathophysiology of shoulder disease, specifically focusing on its influence on the rotator cuff. These inquiries highlight a meaningful correlation between vitamin D levels and the intricate regenerative processes associated with the rotator cuff [6,7]. The benefits of maintaining sufficient vitamin D levels extend to supporting rotator cuff healing, increasing bone mineral density (BMD), and strengthening skeletal muscle [1,8]. Importantly, insufficient vitamin D levels have been empirically demonstrated to hinder the early phases of healing at the rotator cuff repair site, supported by biomechanical and histological data from a rodent model [8].

Additionally, clinical analyses have meticulously examined the role of vitamin D in fatty degeneration and muscle function within the rotator cuff, revealing a significant inverse correlation between serum vitamin D levels and fatty degeneration of the cuff muscle, as well as a positive correlation with isokinetic muscle torque [6].

Currently, there is a pervasive prevalence of vitamin D deficiency in Asian countries, reaching alarming levels of up to 70% [9]. This deficiency seems to be more severe in Asian regions compared to their Western counterparts [10,11]. Despite the noticeable incidence of serum vitamin D deficiency among individuals with rotator cuff tears, there is a discernible lack of investigations scrutinizing the correlation between vitamin D levels and rotator cuff tears within the elderly Thai population. Therefore, this study aims to elucidate the relationship between occurrences of rotator cuff tears and vitamin D levels in the urban Thai elderly demographic. The hypothesis guiding our research suggests that vitamin D deficiency will show an association with the incidence of rotator cuff tears.

## Materials and methods

Study design

This study was a prospective clinical trial conducted at a single tertiary healthcare facility affiliated with Navamindradhiraj University Vajira Hospital, covering the period from July 10, 2022, to July 31, 2023. The research protocol underwent a rigorous approval process by the local Institutional Review Board (IRB), designated as ‘EC’, ensuring meticulous adherence to the ethical principle outlined in the Declaration of Helsinki governing medical research involving human subjects. Following this approval, informed and written consent was diligently obtained from each individual participant duly enrolled in the study.

Recruitment

Our study enrolled participants aged 60 to 80 years, exhibiting unilateral chronic shoulder pain persisting for a duration exceeding three months, clinically attributed to rotator cuff injury and validated through comprehensive clinical assessment. Exclusion criteria were applied to individuals with bilateral shoulder pain, osteoarthritis of the glenohumeral joint, a history of prior shoulder surgery, those aged 80 years or older, individuals currently undergoing vitamin D supplementation within the past month, or with a history of intra-articular injection in the shoulder joint. Additionally, exclusions covered those with hyper- or hypoparathyroidism and individuals with underlying systemic inflammatory conditions such as rheumatoid arthritis. A body mass index (BMI) exceeding 40 kg/m^2^ was also a criterion for exclusion.

Investigations

All participants provided blood specimens in a fasting state, and subsequently, we quantified the calciferol concentration. The reference range for this assay, detailed in the manual, spanned from 11 ng/mL to 70 ng/mL, covering the 2.5th to 97.5th percentiles. The time between blood sample collection and magnetic resonance imaging (MRI) did not exceed a two-week window. Laboratory analyses were conducted at our institution’s Department of Clinical Pathology, with personnel blinded to the study parameters. Vitamin D sufficiency was defined as a serum calcifediol level above 30 ng/mL. Insufficiency between 20 ng/mL and 30 ng/mL, and deficiency below 20 ng/mL [12]. The assessment of fatty degeneration within the supraspinatus, infraspinatus, and subscapularis musculature, tear dimensions, and cartilage thickness was conducted utilizing magnetic resonance imaging (MRI) techniques within the outpatient setting by an orthopedic specialist. Subsequently, all findings were subjected to validation through review by a musculoskeletal radiologist to ensure diagnostic precision and accuracy. Subsequently, the radiological assessment of these findings was undertaken employing the established grading system devised by Goutallier et al. [13]. This system, commonly employed to evaluate fatty infiltration in muscles, particularly within the rotator cuff musculature, delineates five discrete grades: Grade 0, reflective of the absence of fatty infiltration; Grade 1, indicative of the presence of fatty streaks or spots; Grade 2, representing less than 50% fatty infiltration; Grade 3, suggestive of greater than 50% fatty infiltration; and Grade 4, indicative of complete fatty infiltration. Importantly, the radiologists conducting these assessments were unaware of the specific details and objectives of the present study.

Statistical analysis

Statistical analyses employed IBM SPSS Statistics version 22.0 (IBM Corp., Armonk, NY, USA). Continuous variables were presented as mean ± standard deviations and subjected to normality testing using methods such as Skewness-Kurtosis, Histograms, and the Shapiro-Wilk test to determine normal or non-normal distribution. Correlation analyses involving serum vitamin D levels and other variables, including patient age, tear size, severity of rotator cuff issues, BMI, extent of fatty infiltration in the cuff, and cartilage thickness of the humeral head, were conducted using either Person’s or Spearman’s correlation coefficients, based on data type, scale, and distribution normality.

## Results

The analysis of serum vitamin D levels in a cohort of 59 subjects revealed significant findings. Specifically, 20.03% of participants were deficient in vitamin D, 44.07% showed insufficiency, and 33.90% had optimal levels (see Table 1 for detailed data). The mean serum concentration of 25-hydroxyvitamin D was 26.54 ± 8.25 ng/mL.

**Table 1:** Demographic characteristics and distribution of serum vitamin D of the patients

	Sufficiency (>30 ng/mL)	Insufficiency (20-30 ng/mL)	Deficiency (<20 ng/mL)	p-value
Total N (%)	20 (33.90)	26 (44.07)	13 (20.03)	
Gender				0.03
Female	12	6	0	
Male	8	20	13	
Age	67.75 ± 4.54	67.65 ± 5.11	68.08 ± 5.47	0.96
BMI	26.01 ± 3.73	24.95 ± 4.20	24.44 ± 3.44	0.49
Underlying disease	18	18	11	0.06
Degree of fatty infiltration				0.42
0	1	0	0	
I	5	5	2	
II	7	11	4	
III	4	9	3	
IV	3	1	4	
Rotator cuff pathology				0.936
Normal	1	1	0	
Partial-thickness	8	13	7	
Full-thickness	11	12	6	
Tear size	108 (30-360)	60 (40-171)	90 (45-286)	0.829
Superior cartilage thickness	1.70 ± 0.68	1.79 ± 0.51	1.38 ± 0.51	0.363
Middle cartilage thickness	1.68 ± 0.75	1.92 ± 0.77	1.58 ± 0.79	0.981
Inferior cartilage thickness	1.25 ± 0.47	1.27 ± 0.47	1.12 ± 0.58	0.652
Total cartilage thickness	4.63 ± 1.51	4.98 ± 1.24	4.08 ± 1.37	0.669

**Figure 1:**
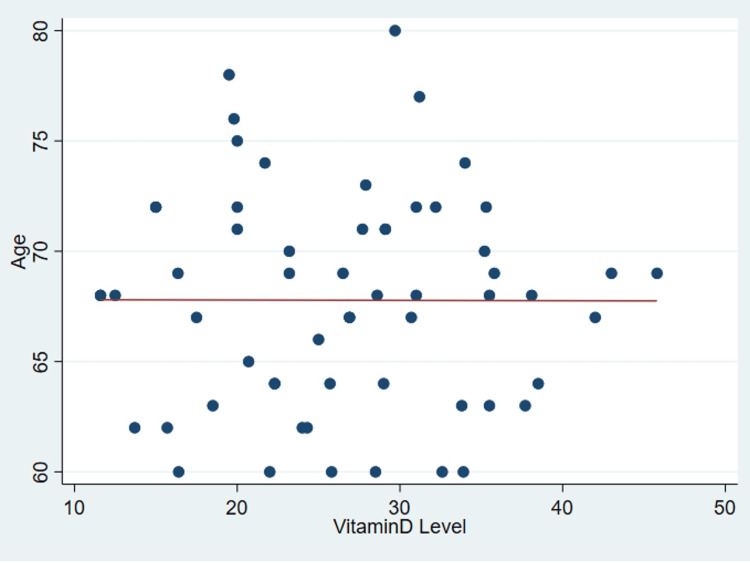
The correlations between age and vitamin D.

**Figure 2:**
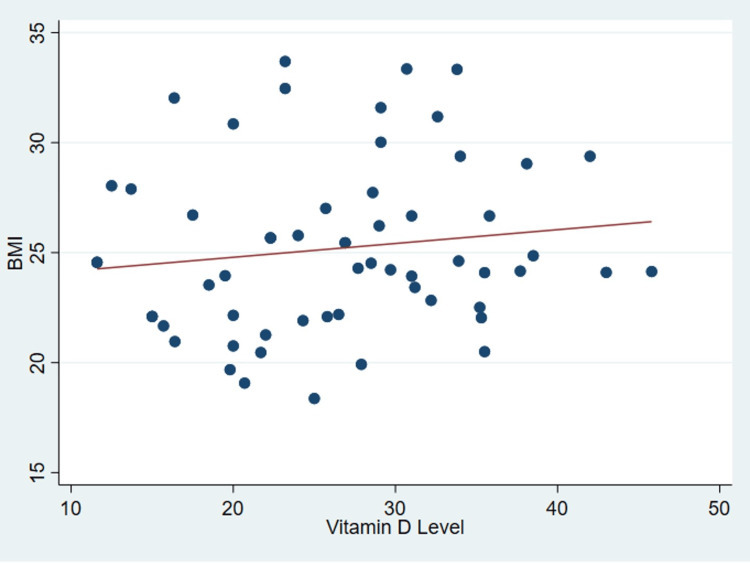
The correlations between body mass index and vitamin D. BMI: Body Mass Index

**Figure 3:**
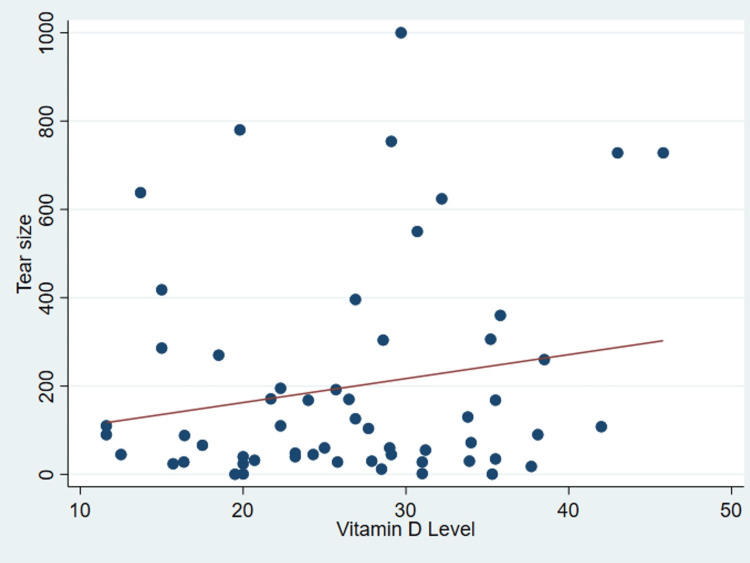
The correlations between tear size and vitamin D.

**Figure 4:**
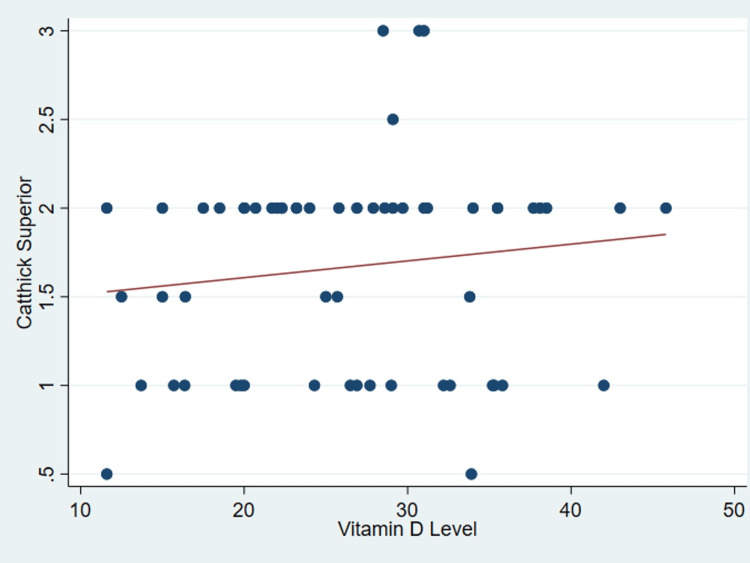
The correlations between cartilage thickness of the superior part of the humeral head and vitamin D. Catthick: Cartilage thickness

**Figure 5:**
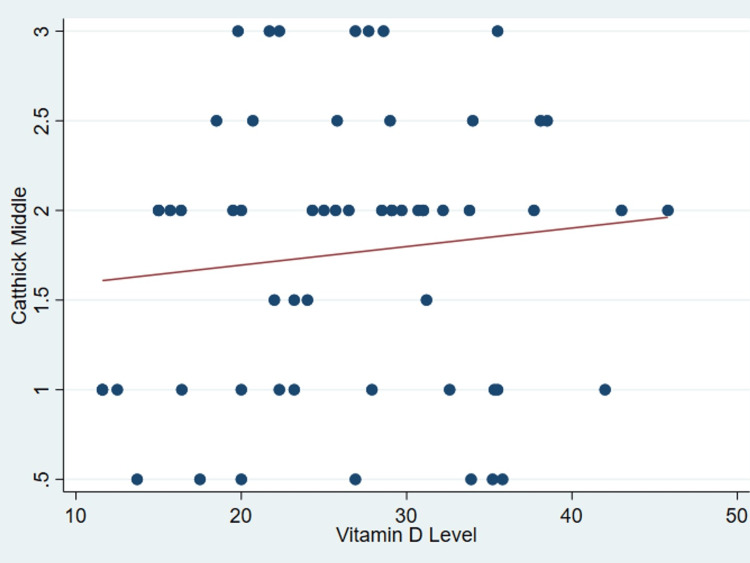
The correlations between cartilage thickness of the middle part of the humeral head and vitamin D. *Catthick: Cartilage thickness

**Figure 6:**
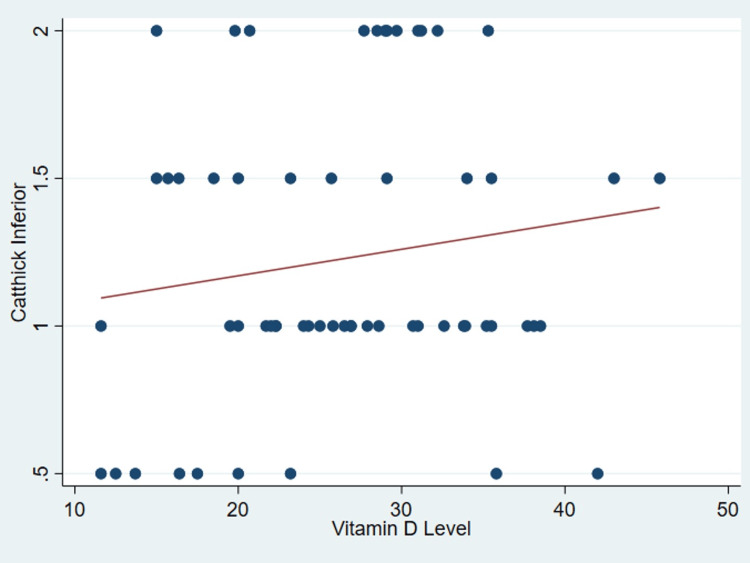
The correlations between cartilage thickness of the inferior part of the humeral head and vitamin D. *Catthick: Cartilage thickness

**Figure 7:**
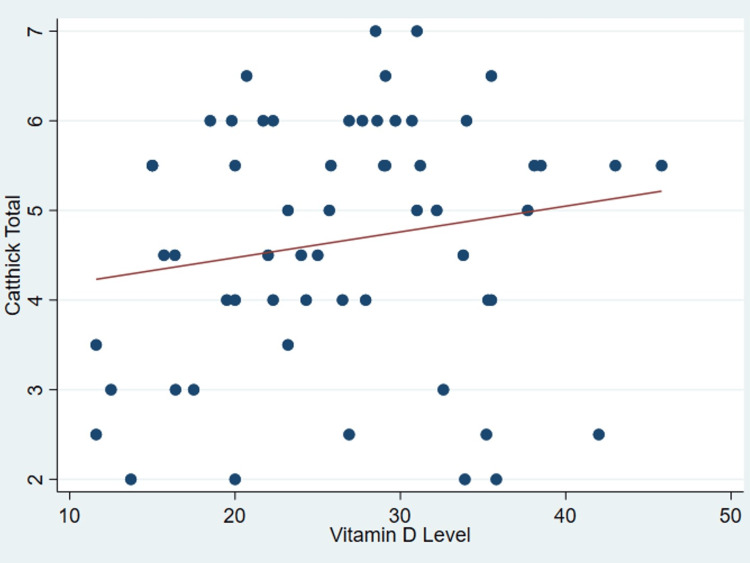
The correlations between cartilage thickness of total parts of the humeral head and vitamin D. *Catthick: Cartilage thickness

**Figure 8:**
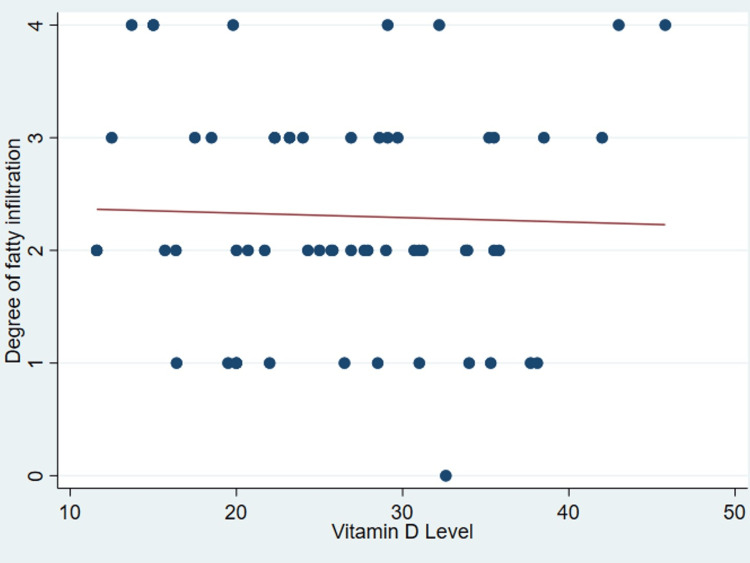
The correlations between fatty infiltration and vitamin D.

**Figure 9:**
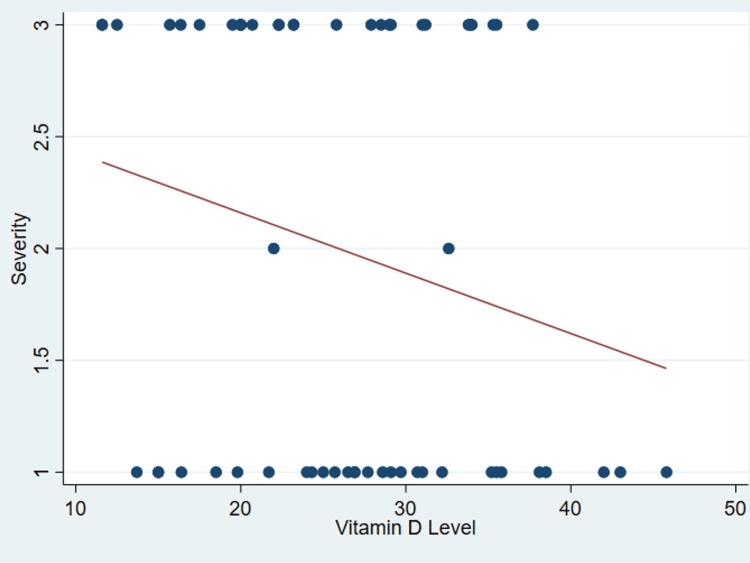
The correlations between severity of rotator cuff tear and vitamin D.

It’s important to note that no discernible correlation was found between serum vitamin D levels and various patient parameters as outlined in Table 2, including age (P = .99) (Figure 1), body mass index (P = 0.31) (Figure 2), tear size (P = 0.41) (Figure 3), and cartilage thickness at different locations on the humeral head (superior, middle, inferior, and total) (P = 0.31, 0.40, 0.26, 0.20, respectively) (Figures 4-7). Additionally, there was no correlation between the degree of fatty infiltration of the rotator cuff (P = 0.81) (Figure 8), and the severity of the rotator cuff condition (P = 0.13) (Figure 9).

**Table 2:** Correlation coefficients (r) between vitamin D level and other characteristics.

Characteristics	Vitamin D level (ng/mL)	P-value
Age	-0.003	0.985
BMI	0.134	0.313
Tear size	0.112	0.408
Superior cartilage thickness	0.134	0.314
Middle cartilage thickness	0.111	0.402
Inferior cartilage thickness	0.15	0.257
Total cartilage thickness	0.171	0.195
Fat infiltration	-0.033	0.805
Severity	-0.198	0.134

However, a strong positive correlation emerged between rotator cuff tear size and both the severity of the rotator cuff condition (P < 0.001) and the degree of fatty infiltration of the cuff (P < 0.001), detailed in Table 3.

**Table 3:** Correlation coefficients (r) between tear size and other characteristics.

Characteristics	Tear size (mm^3^)	P value
Age	0.06	0.64
BMI	0.15	0.28
Vitamin D level	0.11	0.41
Superior cartilage thickness	-0.05	0.73
Middle cartilage thickness	0.13	0.36
Inferior cartilage thickness	0.01	0.95
Total cartilage thickness	0.10	0.45
Fat infiltration	0.70	< 0.001
Severity	-0.72	< 0.001

In contrast, serum vitamin D levels showed no statistically significant differences concerning the severity of the rotator cuff (P = 0.94) and demonstrated no variance among distinct degrees of fatty infiltration of the cuff muscle, as classified by the Goutallier stage (P = 0.42), as determined through Analysis of Variance (ANOVA). Furthermore, a notable gender-based difference in vitamin D levels was observed, with men having higher levels (31.31 ± 6.74) compared to women (25.06 ± 8.18). This difference reached statistical significance (P = 0.01) as determined by the results of the student t-test.

## Discussion

The prevalence of vitamin D deficiency varies significantly across different regions and demographic groups worldwide. In the United States, hypovitaminosis D affects around 40% of the population [14]. Interestingly, in South Asia, approximately 70% or more of the population is affected by vitamin D deficiency, while Southeast Asia displays a wide range of prevalence rates, spanning from 6% to 70% [10]. Previous investigations examining data from 2641 adults aged 15 to 98 across all regions of Thailand have identified the Bangkok population as having the highest prevalence of vitamin D insufficiency and deficiency, at 64.6% and 14.3%, respectively [15]. These findings suggest that younger age is an independent risk factor for inadequate vitamin D levels across both genders [16], contradicting earlier reports that attributed the higher prevalence to aging Thai populations, documented to range from approximately 54% to 77% as of 2011 and 2012 [17,18]. Consistent with existing literature, our own empirical findings support this conclusion, indicating that 20.03% (13 out of 59) of individuals with rotator cuff tears exhibit evidence of hypovitaminosis D.

Hypovitaminosis D has been identified as a contributing factor to various musculoskeletal disorders, such as muscle weakness [19], bone pain [20], and reduced muscle function [19]. Vitamin D plays a crucial role in maintaining muscle strength and integrity. Insufficient levels of this vitamin can lead to muscle weakness, potentially compromising biomechanical stability and increasing the risk of injuries like rotator cuff tears. Moreover, low levels of vitamin D have been linked to impaired muscle regeneration and repair processes. In the context of injuries such as rotator cuff tears, adequate vitamin D levels are essential for optimal healing and rehabilitation of affected muscles. Inadequate vitamin D may hinder the recovery process, raising the likelihood of recurring injury or incomplete recovery. Additionally, vitamin D plays a key role in regulating immune function, and its deficiency has been associated with weakened immune responses. In individuals with rotator cuff tears, diminished immune function due to vitamin D deficiency may impair the body's ability to mount an effective inflammatory and reparative response to the injury [21]. While the precise mechanisms linking hypovitaminosis D and rotator cuff tears are not fully understood, it is clear that optimal vitamin D levels are crucial for maintaining musculoskeletal health, improving muscle function, promoting tissue regeneration, and reducing the risk of injuries, including rotator cuff tears. Therefore, ensuring sufficient vitamin D intake and addressing deficiencies through supplementation or sunlight exposure may provide tangible benefits for both preventing and managing rotator cuff injuries.

Some studies have explored hypovitaminosis D in patients diagnosed with rotator cuff tears. However, research on the prevalence of hypovitaminosis D and its correlation with serum concentration of 25- hydroxyvitamin D within this specific demographic remains limited. Noteworthy contributions to this field include the study conducted by Oh et al. [6], which found a prevalence of 51.3% among patients diagnosed with full-thickness rotator cuff tears, with a mean age of 61.3 years and a mean serum concentration of 44.02 ± 20.26 ng/mL. Similarly, Ryu et al. [22] reported an 88% prevalence among a comparable cohort of patients, with a mean age of 57.5 ± 4.3 years and a serum concentration of 13.82 ± 6.61 ng/mL. Furthermore, Lee et al. [9] documented a prevalence rate of 44.3% among patients with full-thickness rotator cuff tears, with a mean age similar to Ryu et al.'s cohort, accompanied by a serum concentration of 24.70 ± 13.70 ng/mL. In the current investigation, the prevalence of hypovitaminosis D (defined as 25-hydroxyvitamin D levels below 20 ng/mL) in patients afflicted with full-thickness rotator cuff tears, with a mean age of 68.08 ± 5.47 years, was determined to be 20.03%, exhibiting a mean serum concentration of 26.54 ± 8.25 ng/mL. A comparison of the results from this study with previous studies is provided in Table 4.

**Table 4:** A comparison of the prevalence of hypovitaminosis observed in this study with findings from previous studies involving patients diagnosed with rotator cuff injury.

Author/Year	Country	Gender ratio (Male:Female)	Mean age (years)	Prevalence	Mean serum concentration (A comparison of the prevalence of hypovitaminosis from this study with previous studies in patients diagnosed with rotator cuff injury). Vitamin D concentration (ng/mL)
Oh et al./ 2009	Korea	43.4:56.6	61.3	51.3%	44.02 ± 20.26
Ryu et al./ 2015	Korea	47:44	57.5 ± 4.3	88%	13.82 ± 6.61
Lee et al./ 2020	Korea	46.6:53.4	61.9 ± 8.9	44.3%	24.70 ± 13.70
Current study	Thailand	67.9:32.1	67.8 ± 4.9	20.03%	26.54 ± 8.25

Despite diligent investigation, our study failed to establish a significant correlation between vitamin D and other influencing factors. This lack of correlation may be attributed to the limited sample size within our population. However, recent discoveries underscore the irreversibility of fatty degeneration within the cuff muscles, emphasizing its role as a prognostic marker [23,24]. Consequently, it becomes imperative to explore strategies that might mitigate the advancement of this degenerative process, especially among individuals with a pre-existing tear. This could involve surgical interventions where feasible or the use of pharmaceutical supplements. Further investigations are warranted to ascertain the potential correlation between vitamin D levels in the cuff muscle tissue, the extent of fatty degeneration, and serum vitamin D levels. Additionally, these studies should assess whether vitamin D supplementation can lead to substantial improvements in clinical outcomes.

The current investigation is subject to several notable limitations. Firstly, the study’s sample size was relatively modest, encompassing a total of 59 participants. This may introduce the possibility of insufficient statistical power, potentially impeding the detection of correlations between vitamin D and other factors in the study. Secondly, the potential influence of seasonal variations on vitamin D levels was not accounted for in the analysis. This omission may limit the comprehensive understanding of the vitamin’s dynamics within the study context. Thirdly, the exploration of the implications of hypovitaminosis D on the genesis of rotator cuff disease was not undertaken. Consequently, there exists a compelling imperative for further research endeavors aimed at validating the precise role of vitamin D in the etiology of rotator cuff disease or its impact on reparative mechanisms in the context of rotator cuff surgery.

## Conclusions

A negative correlation was identified between serum vitamin D levels and several pivotal parameters associated with rotator cuff pathology within the elderly urban Thai population. These parameters encompassed tear size, fatty infiltration, cartilage thickness, and the severity of rotator cuff tears. However, to consolidate and advance these findings, further empirical inquiry is imperative. The integration of systematic assessments of vitamin D status into the comprehensive management strategies tailored for aging populations afflicted with rotator cuff conditions holds promise for yielding valuable insights into potential therapeutic modalities. Through the conduct of additional empirical investigations and the routine integration of vitamin D assessments into clinical practice, a more nuanced understanding of the nexus between vitamin D levels and the management outcomes of rotator cuff pathology in elderly individuals can be achieved.
